# Associations between elevated uric acid and brain imaging abnormalities in pediatric patients with methylmalonic acidemia under 5 years of age

**DOI:** 10.1038/s41598-024-74710-z

**Published:** 2024-10-14

**Authors:** Mengmeng Du, Shengnan Wu, Yongxing Chen, Shuxian Yuan, Shijie Dong, Huizhen Wang, Haiyan Wei, Changlian Zhu

**Affiliations:** 1 Henan Pediatric Clinical Research Center and Key Laboratory of Child Brain Injury, Institute of Neuroscience, Third Affiliated Hospital of Zhengzhou University, No. 7, Kangfuqian Street, Erqi District, Zhengzhou, China; 2 Department of Endocrinology, Genetics and Metabolism, Henan Children’s Hospital, Children’s Hospital Affiliated to Zhengzhou University, Zhengzhou, China; 3 Department of Radiology, Henan Children’s Hospital, Zhengzhou, China; 4 Center for Brain Repair and Rehabilitation, Institute of Neuroscience and Physiology, University of Gothenburg, Gothenburg, Sweden

**Keywords:** Brain image, Brain structural injury, Children, Methylmalonic academia, Uric acid, Medical research, Neurology

## Abstract

Methylmalonic acidemia (MMA) is the most common inborn organic acidemia, presenting multisystemic complications. Uric acid may have neurotoxic or neuroprotective effects due to its antioxidant or pro-inflammatory properties; however, its role in MMA brain injury remains unclear. We examined the correlation between the serum uric acid levels and brain imaging features of MMA. Data were collected from a cross-sectional study of 216 patients with MMA and 216 healthy matched controls aged 0–5 years in China. Serum uric acid levels were measured, and magnetic resonance imaging and computed tomography findings were retrieved from hospital records. Overall, 74.1% patients had brain abnormalities. Patients in the MMA group with abnormal brain imaging had higher serum uric acid levels than those in the MMA normal brain imaging and control groups. The area under the curve of serum uric acid was 0.74, 0.91, and 0.93 for MMA diagnosis with abnormal brain images, basal ganglia changes, and globus pallidus changes, respectively. Higher serum uric acid levels were independently associated with abnormal brain images. Children aged < 5 years with abnormal brain images in MMA exhibit elevated serum uric acid levels, serving as an effective auxiliary diagnostic indicator and independent risk factor for brain tissue injury.

## Introduction

Methylmalonic acidemia (MMA) is a severe metabolic error of organic acids caused by a defect in the methylmalonyl-CoA mutase enzyme or the synthesis and transport of its cofactor. This enzyme is involved in crucial steps of the catabolic pathways of amino acids (valine, isoleucine, methionine, and threonine), cholesterol side chains, and odd-chain fatty acids^1,2^. The incidence of MMA widely varies worldwide, with a reported incidence in China ranging from 1:65,000 to 1:5,000^3^. Patients with MMA may experience multiple physical system injuries, particularly affecting the central nervous system, leading to high mortality and disability rates^4–6^.

Previous reports have indicated that the radiological presentation of MMA is diverse and non-specific^7^. The primary radiological findings of MMA-related brain injury include changes in the basal ganglia (especially the pallidum) changes^8,9^, myelination delay^10^, ventricular dilation^11^, cortical atrophy^12^, periventricular white matter abnormality^13^, hydrocephalus^14,15^, subcortical white matter abnormality^16^, and thinning of the corpus callosum^17^, amongst others.

Brain damage is common in patients with MMA, and unfortunately, the primary disease of MMA is difficult to diagnose early, and the associated brain injuries are also easily overlooked. The main clinical manifestations of MMA are seizures, developmental delay, cognitive impairment, movement disorders, hypotonia, psychiatric symptoms, and an altered level of consciousness (from lethargy and somnolence to coma)^18,19^. A study found no significant differences in imaging presentation between patients with MMA identified by newborn screening after birth and those clinically diagnosed after exhibiting suspected symptoms^16^, indicating that injury occurs silently in the period before symptom onset. Neuroimaging plays a crucial role in identifying central nervous system damage, facilitating early and accurate diagnosis, which can improve therapeutic efficacy^7^. Another diagnostic approach for MMA-induced brain injury involves neuropsychological assessment tests^20^. Some magnetic resonance imaging (MRI) abnormalities in patients with MMA have been linked to developmental delays in children^16^. However, the results of neuropsychological assessment tests depend on patient compliance. Therefore, it is important to search for serum biomarkers of brain structural injury in MMA and explore the potential sites of injury.

Uric acid is an end-metabolite of adenine and guanine^21^, formed from exogenous purines and endogenously from damaged, dying, and dead cells^22^. As a potent antioxidant, uric acid is responsible for more than half of the free radical scavenging activity of the blood^23^. Recent studies have established a correlation between the serum uric acid levels and the risk of the development and progression of several neurological diseases, including multiple sclerosis^24^, Parkinson’s disease^25^, and Alzheimer’s disease^26^. There is increasing evidence that uric acid is closely associated with inflammation and oxidative stress^23,27,28^. Although the pathophysiology of MMA brain injury remains unknown, oxidative stress, inflammation, and the accumulation of harmful metabolites may play significant roles^19,29^. Häberle proposed that elevated uric acid levels are among the indicators of MMA acute encephalopathy in distinguishing other congenital metabolic disorders^30^. To the best of our knowledge, no previous study has correlated the serum uric acid levels with brain imaging findings in a population of patients aged 0–5 years.

Our primary was to comprehensively investigate the relationship between the serum uric acid levels and the radiological presentations of MMA. Additionally, we aimed to examine uric acid’s potential role as a diagnostic marker for the risk of developing brain injury or for predicting the brain injury regions of MMA, which may pave the way for new diagnostic strategies for MMA.

## Results

### Characteristics of the participants

In total, 216 patients with MMA (128 boys and 88 girls) and 216 healthy controls were included in the study. The general characteristics of the study population are presented in Table 1. There were no significant differences in age or sex among healthy controls, patients with MMA with normal brain images, and patients with MMA with abnormal brain images. Dried blood spot C3 and C3/C2 ratios and urine methylmalonic acid levels increased in patients with MMA.

**Table 1:** Basic features of participants in the study.

Measurement	Healthy controls (n = 216)	MMA (n = 216)		MMA (n = 216)
		Normal brain images (n = 56)	Abnormal brain images (n = 160)	
Male, n (%)	129 (59.7)	38 (67.9)	90 (56.3)	0.213
Age, months	2.8 (1.5–10.2)	2.4 (1.4–8.9)	2.9 (1.4–7.9)	0.982
Serum uric acid, µmol/L	189 (157.1–250.8)	191.8 (149.1–239.1)	265.8 (203.7–334.8)^b, c^	< 0.001
GFR, mL/min	86.1 (66.7–112.1)	86.9 (60.6–119.9)	83.3 (61.6–117.8)	0.858
Creatinine, µmol/L	31 (23.7–38)	27.1 (21.4–37.6)	28.2 (21.4–38.5)	0.270
Urea, mmol/L	4.3 (2.9–5.8)	3.6 (2.7–4.5)	3.7 (2.7–4.7)^b^	0.008
ALT, U/L	26.8 (19.6–34.1)	35.4 (29.4–46.3)^a^	33.8 (24.5–42.3)^b^	< 0.001
AST, U/L	26.5 (21.5–34.2)	40.5 (32.8–53.2)^a^	34.0 (25.7–46.3)^b, c^	< 0.001
Serum Hcy, µmol/L	9.5 (7.4–11.5)	43.9 (8.9–94.9)^a^	77.8 (17.5–127.3)^b^	< 0.001
Plasma ammonia, µmol/L	17.9 (13.9–24.6)	34.2 (19.9–46.5)^a^	35.5 (22.0–52.8)^b^	< 0.001
pH	-	7.4 (7.3–7.4)	7.4 (7.3–7.4)	0.526
HCO^3−^, mmol//L	-	21.7 (19.2–23.2)	20.4 (17.3–23.3)	0.068
AG, mmol//L	-	15.1 ± 4.7	17.4 ± 6.2^c^	0.005
Lactic acid, mmol//L	-	2.5 (1.8–3.1)	2.8 (2.2–3.7)	0.110
Blood spots C3, µmol/L	-	6.5 (4.5–9.3)	6.3 (4.8–8.7)	0.991
C3/C2	-	0.4 (0.3–0.7)	0.5 (0.4–0.7)^c^	0.049
Urine methylmalonic acid (mmol/mmol creatinine)	-	83.8 (49.4–163.3)	82.2 (30.8–199.5)	0.773

AG, anion gap; ALT, alanine aminotransferase; AST, aspartate transaminase; C2, acetylcarnitine; C3, propionylcarnitine; GFR, glomerular filtration rate; Hcy, homocysteine; MMA, methylmalonic acidemia. P-values derived from the Kruskal–Wallis test, Mann–Whitney U test, and Student's t-test analysis are indicated.

^a^p < 0.05, comparison between patients with MMA with normal brain images and healthy controls.

^b^p < 0.05, comparison between patients with MMA with abnormal brain images and healthy controls.

^c^p < 0.05, comparison between patients with MMA with abnormal and normal brain images. Data are presented as means ± standard deviations, medians (25th–75th percentile), or numbers (percentages).

**Fig. 1:**
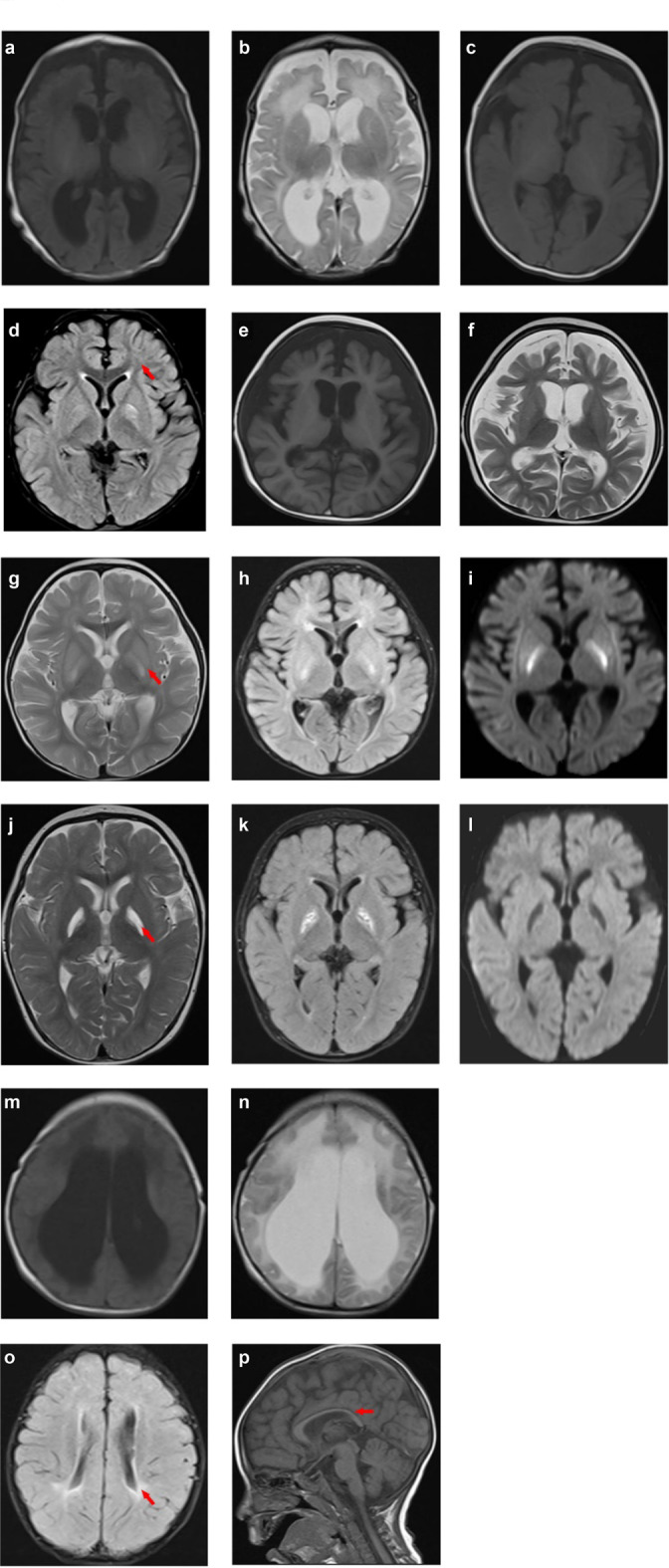
Brain MRI findings. (**a**–**b**) The MRI findings of ventricular dilation were acquired with axial T1-weighted image (**a**) and axial T2-weighted image (**b**). (**c**) The MRI of a 4-month-old boy revealed myelination delay, with relatively weak hyperintensity in the internal capsules in the axial T1-weighted image. (**e**–**f**) The MRI images of cerebral atrophy were acquired with axial T1-weighted image (**e**) and axial T2-weighted image (**f**). (**g**–**l**) The MRI findings revealed basal ganglia changes (**g**–**i**) and globus pallidus changes (**j**–**l**), and the axial T2-weighted (**g**, **j**) and T2-FLAIR (**h**, **k**) images showed symmetric hyperintense signals; the diffusion-weighed images showed high (**i**) and low signals (**l**), respectively. (**m**–**n**) The MRI findings of hydrocephalus were acquired with axial T1-weighted image (**m**) and axial T2-weighted image (**n**). (**o**) The axial T2-weighted MRI showed periventricular white matter abnormality. (**p**) The sagittal T1-weighted showed thin corpus callosum. MRI, magnetic resonance imaging

**Fig. 2:**
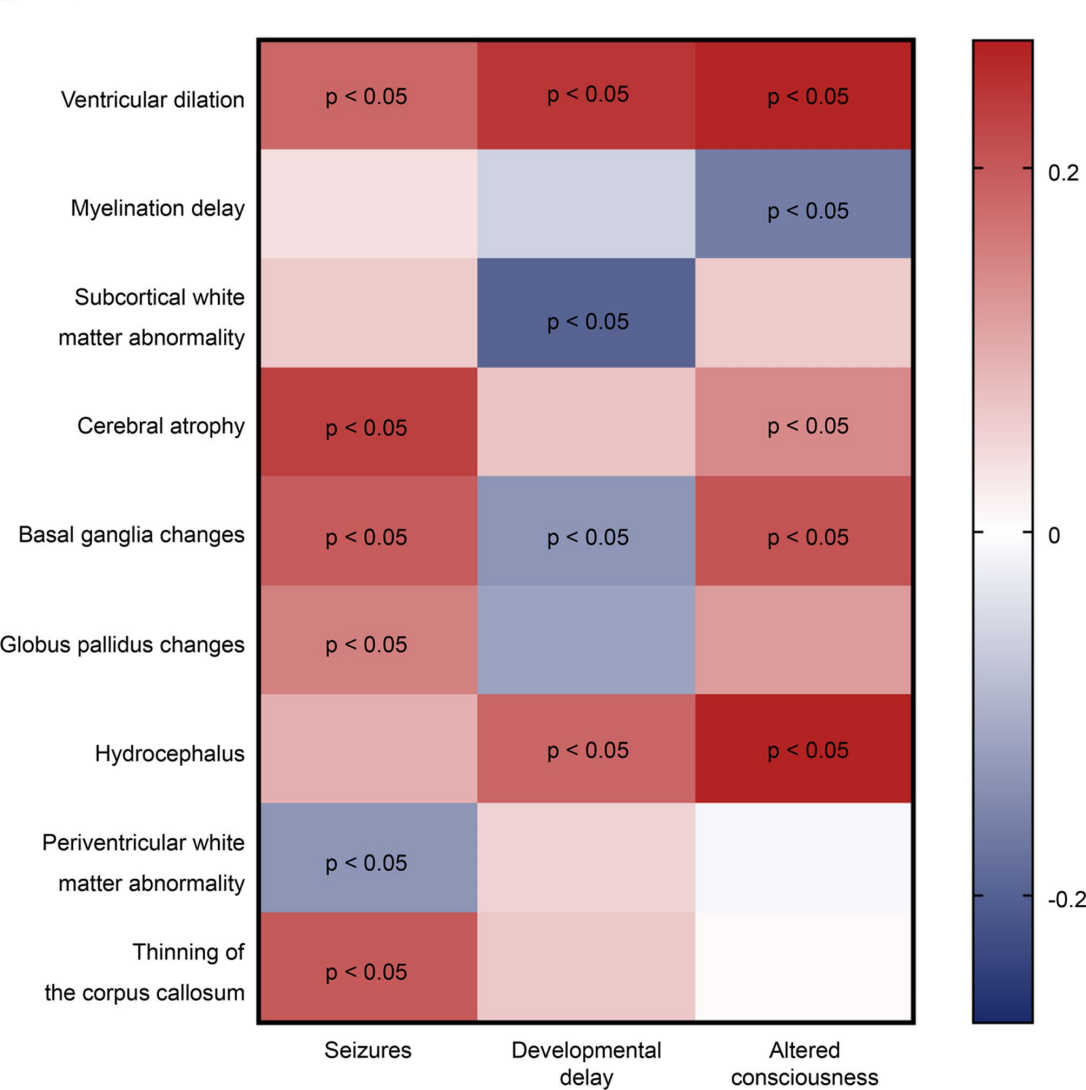
Association of various types of abnormal brain imaging with neurological symptoms. Spearman correlation analysis was performed to investigate the associations between different types of abnormal brain imaging findings and neurological symptoms in patients with MMA. A redder or bluer color indicates a higher correlation. The p-values derived from the Spearman correlation analysis are presented.

The most common brain imaging findings in patients were ventricular dilation, myelination delay, subcortical white matter abnormalities, cerebral atrophy, and basal ganglia changes (Fig. 1). In this study, 128 patients developed neurological symptoms, including seizures, developmental delays, and altered consciousness, while 88 patients did not; these patients were divided into the positive and negative groups. The incidence of abnormal images, especially ventricular dilation, cerebral atrophy, basal ganglia changes, and hydrocephalus, was significantly higher in the neurological symptoms positive group than in the negative group (p < 0.05). The number and frequency of the findings are presented in Table 2. As shown in Fig. 2, ventricular dilation was correlated with seizures, developmental delay, and altered consciousness (p < 0.05). Cerebral atrophy and basal ganglia changes were correlated with seizures and altered consciousness (p < 0.05). Globus pallidus changes and thinning of the corpus callosum were correlated with seizures (p < 0.05). Hydrocephalus was correlated with developmental delay and altered consciousness (p < 0.05).

**Table 2:** Differences in brain image findings in patients with MMA with and without neurological symptoms.

Brain image findings (N)	Present neurological symptoms (N, %)		c2	p-value
	Positive (128)	Negative (88)		
Abnormal image (160)	108(84.4)	52 (59.1)	17.359	< 0.001
Ventricular dilation (82)	65 (50.8)	17 (19.3)	21.919	< 0.001
Myelination delay (68)	36 (28.1)	32 (36.4)	1.641	0.200
Subcortical white matter abnormality (63)	35 (27.3)	28 (31.8)	0.505	0.477
Cerebral atrophy (53)	40 (31.3)	13 (14.8)	7.646	0.006
Basal ganglia changes (38)	28 (21.9)	10 (11.4)	3.974	0.046
Globus pallidus changes (28)	19 (14.8)	9 (10.2)	0.985	0.321
Hydrocephalus (35)	31 (24.2)	4 (4.5)	14.865	< 0.001
Periventricular white matter abnormality (23)	14 (10.9)	9 (10.2)	0.028	0.868
Thinning of the corpus callosum (6)	5 (3.9)	1 (1.1)	-	0.405

### The serum uric acid levels of MMA

**Fig. 3:**
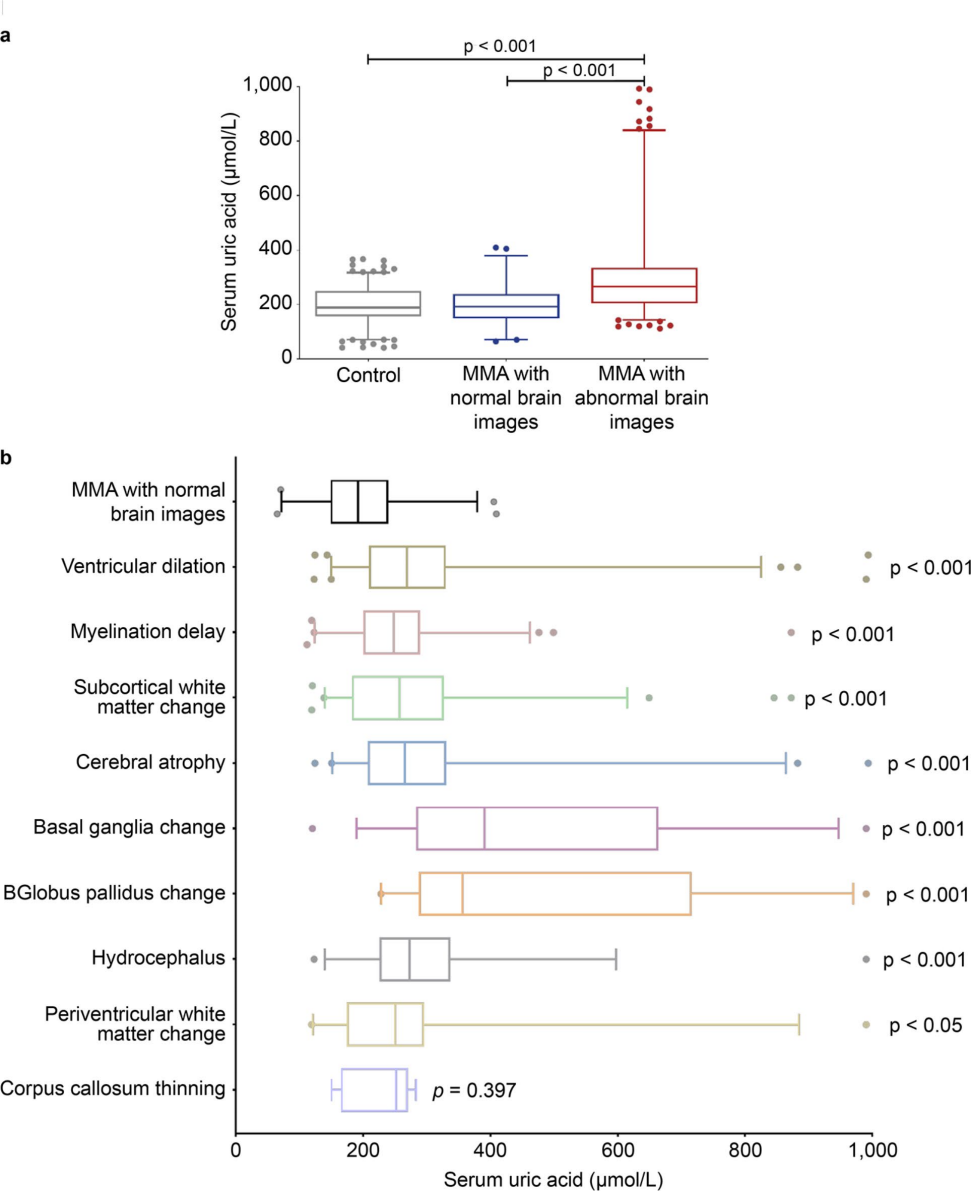
Serum uric acid levels in each group. (**a**) Comparison of the serum uric acid levels among healthy controls, patients with MMA with normal brain images, and those with abnormal brain images. (**b**) Comparison of the levels of serum uric acid between the various types of abnormal brain imaging and normal brain imaging in MMA. The p-values derived from the Kruskal–Wallis test and Mann–Whitney U test analyses are indicated. MMA, methylmalonic acidemia.

Among the 216 patients, 160 (74.1%) had abnormal brain imaging findings. The serum uric acid levels in the MMA abnormal brain images group were significantly higher than those in the MMA normal brain images and control groups (p < 0.001). However, no difference was observed between the MMA normal brain imaging group and the control group (p > 0.05). The levels of serum uric acid in patients with ventricular dilation, myelination delay, subcortical white matter abnormalities, cerebral atrophy, basal ganglia changes, globus pallidus changes, hydrocephalus, and periventricular white matter abnormalities were significantly higher in the MMA normal brain imaging group (p < 0.05; Table 1, Fig. 3).

### Receiver operating characteristic (ROC) analysis of serum uric acid

**Fig. 4:**
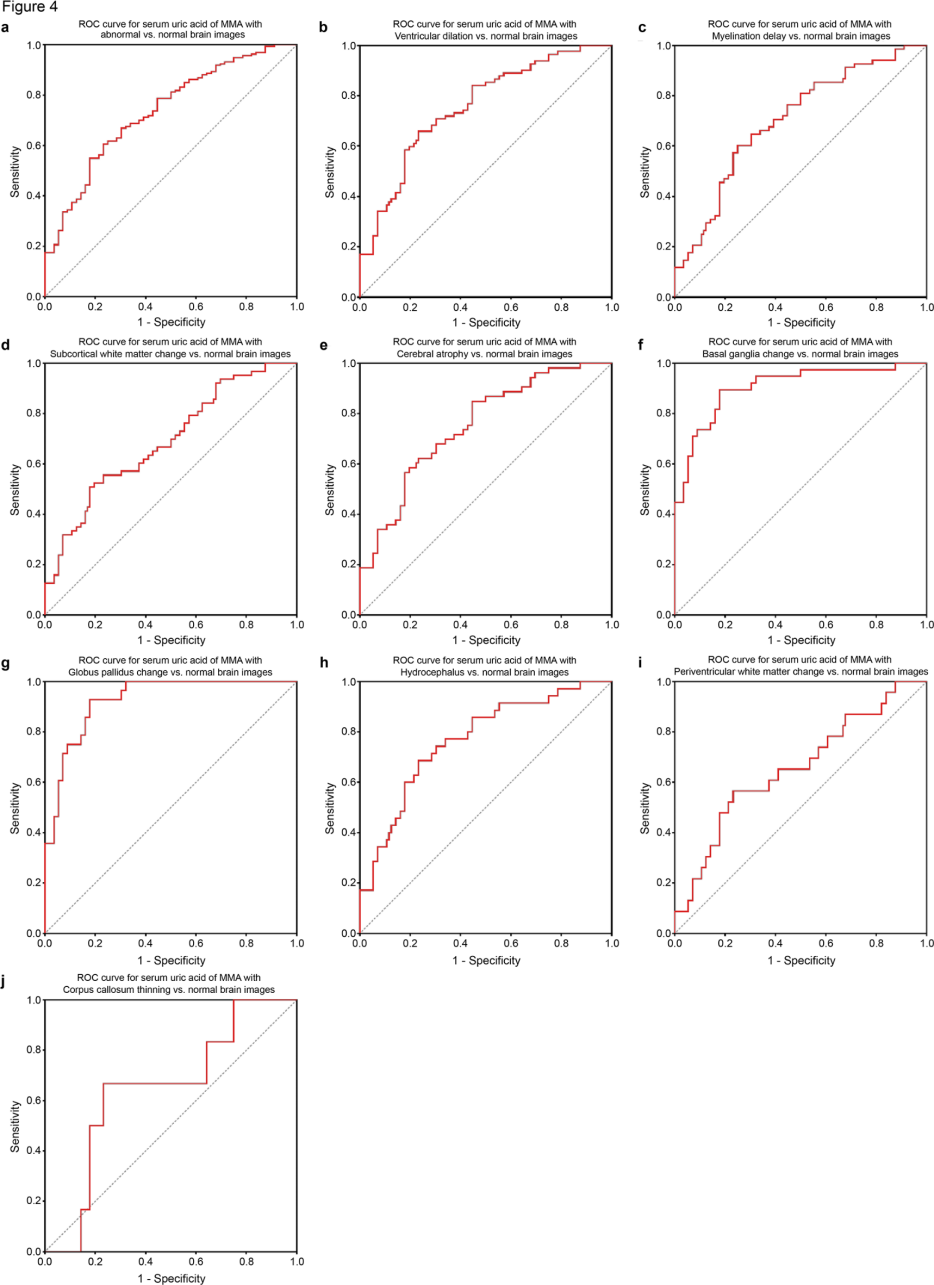
ROC analysis of serum uric acid for MMA brain images. ROC analysis for predicting MMA with abnormal brain images (**a**), ventricular dilation (**b**), myelination delay (**c**), subcortical white matter abnormality (**d**), cerebral atrophy (**e**), basal ganglia changes (**f**), globus pallidus changes (**g**), hydrocephalus (**h**), periventricular white matter abnormality (**i**), and thinning of the corpus callosum (**j**). MMA, methylmalonic academia; ROC, receiver operating characteristic.

Figure 4 and Table 3 illustrate the ROC curve analysis of the serum uric levels for predicting abnormal brain images in patients with MMA. The results of the ROC analysis, including the area under the curve (AUC), cutoff values, p-values, sensitivity, specificity, and positive and negative predictive values, are summarized in Table 3. The AUC of the serum uric acid for diagnosing MMA with abnormal brain images was 0.74 (95% confidence interval [CI]: 0.66–0.81) (p < 0.001). The cutoff value for detecting abnormal MMA brain images was 240.45 µmol/L.

**Table 3:** Coordinates of ROC for serum uric acid to discriminate patients with MMA with abnormal brain images.

Imaging findings	AUC (95% CI)	p-value	Cutoff values	Sensitivity, %	Specificity, %	PPV, %	NPV, %
All imaging abnormality	0.74 (0.66–0.81)	< 0.001	240.45	60.6	76.8	88.2	40.6
Ventricular dilation	0.75 (0.67–0.84)	< 0.001	240.45	65.9	76.8	80.6	60.6
Myelination delay	0.70 (0.61–0.79)	< 0.001	237.75	60.3	75	75.9	61.4
Subcortical white matter abnormality	0.69 (0.60–0.78)	< 0.001	253.9	50.8	82.1	78	60.3
Cerebral atrophy	0.75 (0.66–0.84)	< 0.001	197.7	84.9	55.4	64.3	79.5
Basal ganglia changes	0.91 (0.84–0.97)	< 0.001	256.65	89.5	82.1	77.3	92
Globus pallidus changes	0.93 (0.87–0.98)	< 0.001	256.65	92.9	82.1	72.2	95.8
Hydrocephalus	0.77 (0.67–0.87)	< 0.001	240.7	68.6	76.8	64.9	83.3
Periventricular white matter abnormality	0.66 (0.52–0.79)	0.031	243.05	56.5	76.8	43.5	76.8
Thinning of the corpus callosum	0.65 (0.43–0.86)	0.243	244.4	66.7	76.8	23.5	95.6

AUC, area under the ROC curve; CI, confidence interval; MMA, methylmalonic acidemia; NPV, negative predictive value; PPV, positive predictive value.

ROC analysis of specific abnormal brain images revealed that the AUC of serum uric acid for diagnosing basal ganglia changes was 0.91 (95% CI: 0.84–0.97), while that for globus pallidus changes was 0.93 (95% CI: 0.87–0.98). The cut-off value for basal ganglia and globus pallidus changes was 256.65 µmol/L, with corresponding sensitivities of 0.895 and 0.929, and specificities of 0.821 and 0.821, respectively. The serum uric acid levels were not used for diagnosing other abnormal brain images.

### Factors affecting MMA brain structural injury

In total, 216 patients with MMA were analyzed using multivariate logistic regression analysis for structural brain injury (Table 4). The serum uric acid levels were stratified according to the optimal cutoff value of 240.45 µmol/L determined by ROC analysis. After adjusting for age and sex (Model 1), serum uric acid levels > 240.45 µmol/L was independently associated with abnormal brain images in patients with MMA (odds ratio [OR]: 5.88, 95% CI: 2.44–14.2, p < 0.001). Further, adjusting for lactic and glomerular filtration rate (GFR) in Model 2 showed significant relationships (OR: 5.7, 95% CI: 2.31–14.02, p < 0.001).

**Table 4:** Multivariate logistic regression analysis of predictors for MMA abnormal brain image.

		OR (95% CI)	p-value
Crude	Uric Acid ≥ 240.45 µmol/L vs. < 240.45 µmol/L	5.09 (2.54–10.22)	< 0.001
Model 1	Crude + Age + Sex	5.88 (2.44–14.2)	< 0.001
Model 2	Model1 + lactic acid + GFR	5.7 (2.31–14.02)	< 0.001

Multivariate logistic regression analysis. CI, confidence interval; GFR, glomerular filtration rate; OR, odds ratio; vs., versus.

## Discussion

To the best of our knowledge, this is the first study to systematically correlate the serum uric acid levels with brain imaging findings in a population of patients aged 0–5 years. Children with abnormal MMA brain imaging findings have significantly higher serum uric acid levels than those with normal brain imaging findings. The uric acid levels were elevated in children with abnormal brain imaging findings, except for corpus callosum thinning. According to the comprehensive analysis of the ROC curve and multivariate logistic regression of this study, serum uric acid levels exceeding 240.45 µmol/L may indicate abnormal brain imaging, prompting further confirmatory brain imaging examination. Due to the good diagnostic value of serum uric acid for abnormal changes in basal ganglia and globus pallidus, a uric acid concentration of 256.65 µmol/L could potentially serve as a suitable cutoff value for use as a diagnostic biomarker.

MMA, also known as methylmalonic aciduria, is caused by the presence of a defective methylmalonyl-CoA mutant enzyme or its coenzyme, cobalamin. This leads to the accumulation of methylmalonic acid and its associated organic acids, causing damage to multiple body systems, especially the nervous system^18^. A meta-analytical study of 111 articles on MMA reported a global prevalence of 1.14 per 100,000 newborns and 652.11 per 100,000 clinically suspected patients^31^. Additionally, the prevalence of MMA has increased in recent years. China has a higher prevalence of MMA compared to other regions of the world^32^. Although the incidence of neurological complications is unknown, neurological injuries are common and are associated with high mortality and disability rates. In a study involving 64 patients with MMA, 73% had cognitive impairment^33^. In our study, 216 patients with MMA were enrolled, and 74.1% presented with abnormal brain images.

MMA is a rare disease with a complex clinical phenotype and genotype and is difficult to identify from signs and symptoms alone^18^. Similarly, MMA brain injury has diverse and non-specific clinical manifestations with symptoms, such as seizures, poor feeding, developmental delays, lethargy, and loss of consciousness^7^. Neuroimaging is not routinely performed for patients with MMA. Neuroimaging examinations are performed only after significant signs of neurological impairment have been demonstrated and to further rule out congenital or acquired abnormalities of the nervous system. Cognitive impairment is usually diagnosed through neuropsychological assessment tests^20^, which, depend on patient compliance. Children aged < 5 years or those in a metabolic crisis may be unable to follow instructions or complete these tests due to their age or illness. Therefore, identifying serum biomarkers that could serve as early indicators of MMA brain injury would be beneficial. Such biomarkers could facilitate earlier diagnosis and timely intervention, potentially preventing irreversible brain damage and reducing the need for extensive diagnostic procedures.

In this study, anomalous brain images were prevalent among patients with MMA. The most common imaging abnormalities included ventricular dilatation, myelination delay, subcortical white matter abnormalities, cerebral atrophy, basal ganglia (including globus pallidus) abnormalities, hydrocephalus, and periventricular white matter abnormalities. Although neurological related symptoms were associated with these abnormal brain images, the correlation was relatively weak. Children with abnormal brain imaging findings of MMA present relatively high uric acid levels. Under conditions of reduced cellular oxygen supply, uric acid is produced via the oxidation of hypoxanthine and is excreted via enzymatic pathways which are regulated by metabolic products and free radicals^21^. Therefore, high uric acid levels have been suggested as a marker of cellular oxygen deprivation. Under normal conditions, methylmalonyl-CoA generates succinyl-CoA, which participates in the tricarboxylic acid cycle. When the conversion of methylmalonyl-CoA is blocked, an increase in methylmalonic acid and its associated organic acids results in tricarboxylic acid circulation disorders^34^ and mitochondrial dysfunction^35^, resulting in brain damage. This could be the pathophysiological mechanism underlying high uric acid levels in MMA brain injury. The basal ganglia, especially the globus pallidus, are highly metabolically active and symmetrically affected by metabolic abnormalities in patients with MMA, with images resembling tissue edema caused by cellular hypoxia^11^. A previous spectroscopic research found that patients with MMA have reduced amounts of N-acetyl aspartate and elevated lactate levels in the basal ganglia^36^. The former suggests neuronal damage, and the latter indicates that energy generation is dominated by anaerobic co-lysis. This explains the elevated uric acid levels in the basal ganglia and globular pallidus lesions of patients with MMA in our study. In light of the increased uric acid levels associated with brain injury, interventions such as reducing the intake of purine-rich foods, treatments that enhance oxygen supply and reduce oxidative stress, and medications that promote uric acid excretion and metabolism may be beneficial in managing MMA-related brain injury.

Our analyses demonstrated that a serum uric acid concentration exceeding 240.45 µmol/L is an independent risk factor for abnormal brain imaging of MMA. Serum uric acid levels are regulated by dietary purine intake and renal uric acid excretion^21^. Preschoolers’ diets are relatively simple, and the effects of food on uric acid levels are small. Lactic acid is the substrate for urate transporter-1, a urate exchanger in the proximal tubule that reabsorbs the bulk of filtered urate from the glomerular ultrafiltrate by exchanging luminal urate with monovalent intracellular anions (such as lactate)^37^. Increased lactic acid in patients with MMA results in hyperuricemia owing to increased urate reabsorption; thus, lactic acid shares the propensity to increase serum uric acid levels. Admittedly, uric acid clearance and excretion in early childhood are higher than adult norms and decrease progressively with advancing age^38^. Several studies have suggested that the effects of uric acid on the brain vary between sexes, as female have a better oxidative balance than male individuals^39^. However, some studies have found little difference in uric acid levels between children of different ages and sexes before puberty^40,41^. After adjusting for possible confounders, such as sex, age, lactate level, and GFR, uric acid could be considered a stable biomarker for predicting brain injury risk in children with MMA.

This study had several strengths. The major strength was the comprehensive analysis of clinical data from 216 patients with MMA and controls at a single center in central China. We evaluated the serum uric acid levels in healthy children, patients with MMA with normal brain images, and those with abnormal brain images. This allowed us to propose cut-off values for abnormal uric acid levels, assessing the utility of this measure to assist in the diagnosis and discovery of brain injury in MMA. Additionally, we employed diverse statistical methods to examine the utility of serum uric acid as a biomarker for predicting the risk of brain structural injury in MMA.

However, our study had some limitations. First, some confounders, such as lifestyle, region, and external temperature, were not uniform, and selection bias was present, given that the information was only obtained from individuals at a single center. Second, we were unable to analyze the correlation between brain injury severity, disease duration, and serum uric acid levels because brain imaging was performed using different scan parameters and CT or MRI machines. Unfortunately, this uncertainty will remain an issue until MMA becomes a routine neonatal screening program and pre-symptomatic brain imaging becomes part of the routine clinical management of MMA. This survey did not specifically address multislice proton MR spectroscopy in MMA, which is a technique reflecting the lactate levels in the basal ganglia. Third, we were unable to explain the cause-and-effect relationship between elevated uric acid levels and brain injury. Therefore, further prospective intervention studies are needed to demonstrate whether lowering serum uric acid levels is important for preventing brain injury.

In summary, this study demonstrates that elevated serum uric acid levels are a significant independent risk factor for abnormal brain imaging findings in children with MMA, particularly in regions like the basal ganglia, including the globus pallidus. Serum uric acid levels exceeding 240.45 µmol/L could serve as a valuable biomarker for early detection and risk assessment of brain injury in MMA, guiding further diagnostic imaging and interventions. These findings underscore the potential benefits of routine monitoring and management of uric acid levels through dietary and pharmacological means to mitigate brain injury. Future research should explore the causative relationship between uric acid and brain injury severity and evaluate therapeutic strategies for lowering uric acid levels to improve outcomes in children with MMA.

## Methods

### Study participants

This retrospective study collected data on 216 patients with MMA aged < 5 years who visited Henan Children’s Hospital between 2014 and 2022. Eligible patients were defined by elevated blood propionylcarnitine (C3) levels (> 3.5 μmol/L), the C3/acetylcarnitine (C3/C2) ratio (> 0.2 μmol/L), and urinary methylmalonic acid levels (> 3.6 mmol/mmol creatinine). A total of 216 healthy individuals of similar age were identified from the Health Check database. Informed consent was obtained from the patients’ guardians, and the study protocol was approved by the Medical Ethics Committee of Children's Hospital Affiliated to Zhengzhou University (protocol code 2023-k-124).

### Clinical data acquisition

We collected the clinical data and laboratory test results for each participant. Data were obtained from the children’s hospital records. The brain images of the patients were retrieved, and 216 studies (164 MRI and 52 computed tomography [CT]) were obtained. Each brain image was evaluated by a neuroradiologist with more than 10 years of experience who was unaware of the established diagnosis. The CT scan consisted of 10-mm-thick contiguous axial slices, and the MRI was performed using a 1.5-T MR scanner. Both imaging modalities were evaluated for ventricular, sulci, and fissure prominence, increased white matter T2 signal, and delayed myelination based on a gross visual assessment and simplification scheme. The results were defined by neuroanatomical characterization. Fasting serum uric acid levels were measured using an enzymatic method with a Beckman Coulter AU5800 (Beckman, Brea, CA, USA) automated immunochemistry analyzer. The GFR was calculated using Schwartz’s formula.

### Statistical analysis

The Kolmogorov–Smirnov test was used to determine the data parameter distribution. Student's t-test, Mann–Whitney U-test, or Kruskal–Wallis H-test were used to investigate the differences in the covariates of the continuous data. The chi-squared test and Fisher's exact test were used to investigate differences in brain image findings between patients with MMA with and without neurological symptoms. Correlations were evaluated using Spearman’s method. The contribution of the independent variables to the abnormal brain images of MMA was calculated using multiple linear regression. The predictive value of serum uric acid levels for abnormal brain images of MMA was analyzed using ROC curve analysis. Data were analyzed using SPSS, version 26.0 (IBM Corp., Armonk, NY, USA). Statistical significance was set at p < 0.05.

## Data Availability

The datasets used and/or analysed during the current study are available from the corresponding author on reasonable request.

## Abbreviations

MMA: Methylmalonic academia
GFR: Glomerular filtration rate
ROC: Receiver operating characteristic
